# Global Neurotrauma Surveillance: Are National Databases Overrated? Comment on "Neurotrauma Surveillance in National Registries of Low- and Middle-Income Countries: A Scoping Review and Comparative Analysis of Data Dictionaries"

**DOI:** 10.34172/ijhpm.2022.7577

**Published:** 2022-12-12

**Authors:** Marissa A. Boeck, Hussein Ssenyonjo, Olive C. Kobusingye

**Affiliations:** ^1^Department of Surgery, Zuckerberg San Francisco General Hospital, University of California San Francisco, San Francisco, CA, USA.; ^2^Faculty of Neurosurgery, College of Surgeons of East Central and Southern Africa, Mulago National Referral Hospital, Kampala, Uganda.; ^3^Department of Disease Control and Environmental Health, School of Public Health, College of Health Sciences, Makerere University, Kampala, Uganda.

**Keywords:** Neurotrauma, Registry, Public Health, Surveillance, Low Resource Setting, Injury

## Abstract

Injuries are a public health crisis. Neurotrauma, a specific type of injury, is a leading cause of death and disability globally, with the largest burden in low- and middle-income countries (LMICs). However, there is a lack of quality neurotrauma-specific data in LMICs, especially at the national level. Without standard criteria for what constitutes a national registry, and significant challenges frequently preventing this level of data collection, we argue that single-institution or regional databases can provide significant value for context-appropriate solutions. Although granular data for larger populations and a universal minimum dataset to enable comparison remain the gold standard, we must put progress over perfection. It is critical to engage local experts to explore available data and build effective information systems to inform solutions and serve as the foundation for quality and process improvement initiatives. Other items to consider include adequate resource allocation and leveraging of technology as we work to address the persistent but largely preventable injury pandemic.

 Injuries are a public health crisis. In 2019 over four million deaths and nearly 250 million disability-adjusted life years (DALY), just under 10% of the global DALY burden, were due to trauma. Unintentional injuries, transport injuries, and self-harm and violence fell within the top 20 causes of death and DALY’s across age and sex globally, and within the top 10 causes of deaths and DALYs for those aged 5-69 years and 15-49 years, respectively. Injuries due to self-harm and violence, and transportation are a leading cause of death in men aged 15-49 years worldwide.^1^ Yet all the above are estimates that rely on and are susceptible to inconsistent data sources and modeling. As highlighted by Barthélemy et al,^2^ neurotrauma, a specific type of injury, is itself a leading cause of death and disability globally, with the largest burden in less resourced settings. Even more challenging to accept is that most of these incidents are preventable with appropriate, evidence-based interventions. Just like other public health emergencies, such as the current coronavirus disease 2019 (COVID-19) pandemic, traumatic injuries require a similar focus and commitment.

 Public health teaches us that the first steps to addressing a problem are to measure its magnitude and assess risk and protective factors. Yet there are many obstacles to accomplishing these first steps for trauma, and especially neurotrauma, since most places do not routinely collect data on these diagnoses, and especially not at a national level. Barthélemy et al found 15 low- and middle-income countries (LMICs) with 16 national trauma registries tracking neurotrauma-specific data elements.^2^ Although the authors consider this to be a low number, these results surpass those found in a 2019 review that found of 65 distinct LMIC trauma registries, only seven were multi-hospital and 66% (N=43) were on-going versus short-term registries for a fixed time period.^3^ Data elements were not reviewed in detail so it is unclear which registries in the 2019 review collected the neurotrauma elements explored by Barthélemy et al. Also, the discrepancy between national registries found between these two studies supports the ambiguity around what criteria designate a registry as national. Although a larger, more nationally representative registry is an ideal worth working towards, this expectation may be too high of a bar, especially for resource-limited settings, while local and regional registries can provide a glimpse into a country’s neurotrauma burden. Furthermore, criteria for a registry to be considered national are not standardized. Although originating from a high-income country, an example is the National Trauma Databank (NTDB) in the United States and Canada, which was started in 1989 and relies on the National Trauma Data Standard to dictate collected variables and data format.^4^ In 2019 the NTDB had participation from 764 hospitals across the United States and Canada,^5^ which prior reports noted to potentially represent one-third or less of hospitals treating trauma patients in certain regions without comment of their ability to represent the whole.^6^ Despite this limitation, the NTDB is considered a gold standard for national trauma registries globally. The composite dataset, as well as smaller data subgroups, even at the individual hospital level, serve as the basis for quality improvement initiatives. We should have similar realistic expectations and practices for lower-resourced settings.

 The authors took an innovative approach for this review, not only performing a literature review and convenience sampling of select researchers across multiple languages, but also cold contacting ministries of health and global neurotrauma organizations to find additional registries. Although the latter method was not fruitful in this instance, it recognizes that many activities are likely happening in-country, either locally or nationally, that are not reflected in the published literature. An additional observation is that the contacted neurotrauma organizations have a global focus and are mainly based in higher-resourced settings, and makes an assumption that all neurotrauma registry efforts would be known by these international organizations.^7-9^ National and regional surgical associations and colleges are a great resource that likely would have more knowledge of local efforts, like the College of Surgeons of East, Central and Southern Africa or the Neurosurgical Society of Uganda. Another option would be to search the gray literature, along with in-country hospital and public health organization websites, to explore other ongoing surveillance mechanisms, such as exists in Bolivia with their National Statistics Institute.^10^

 Besides issues with incomplete sampling of injured patients in a registry, whether local, regional, or national, there is large variability in data quantity and quality in the neurotrauma registries reviewed as compared to the gold standard the authors selected: the World Health Organization’s (WHO’s) minimum dataset for injury from the International Registry for Trauma and Emergency Care.^11^ An absence of quality data can limit a location’s ability to efficiently allocate resources, develop interventions based on local disease burden and needs, and pursue quality improvement initiatives. As mentioned by the authors, incidence and prevalence estimates for diseases are frequently inaccurate due to this lack of data – so while certain countries and regions may appear to have lower rates of neurotrauma, it is potentially due to data gaps rather than an actual lack of pathology. Modeling aims to fill this gap but can have its own issues, where limited data and imperfect proxies can lead to uncertain and unstable estimates. Regarding quality, a globally accepted minimum dataset is essential for cross-registry comparison and benchmarking, with various minimum dataset versions that can be adapted depending on available resources. This issue is not unique to neurotrauma registries and is a struggle amongst all trauma registries, if not any surveillance tool. While the WHO International Registry for Trauma and Emergency Care minimum dataset, developed over many years with stakeholder and expert input, aims to serve as a reference,^11^ it is still a new resource and not widely applied. Historically there have been discussions with the American College of Surgeons to provide free global access to the NTDB, based on the National Trauma Data Standard – however the variables required for participation in the registry do not reflect routinely performed tests or available data in many LMICs. This includes the Injury Severity Score, which relies on specific imaging, such as computed tomography, for accurate scoring, a technology that is not routinely available or utilized in many LMICs.

 Challenges with sustained, reliable registries for disease surveillance are not new, especially in resource-denied settings, as highlighted in the current review. Frequently cited barriers include funding, staffing, stakeholder engagement, data completeness, quality, and dissemination, and infrastructure.^12^ Facilitators include identifying a local registry champion, creating a minimum dataset that only focuses on necessary data to minimize the collection burden, providing adequate financing and training, ensuring data quality, and implementing feasible data collection methods.^12,13^

 Obstacles touched upon by Barthélemy et al in their discussion is a lack of adequate workforce, infrastructure, policy, and financing for registries in the face of staggering clinical volume.^2^ Throughout the published literature and from our experience, there is frequently a reliance on busy clinical hospital staff to do registry data collection in real-time, either in paper or electronic format. This practice is frequently justified by the lack of a reliable medical record to retrospectively extract data from and the need to collect the data prospectively at the time of the patient encounter. Yet this practice begs the question why we expect more from resource-denied settings and people that work within them than from higher resourced locations. At Zuckerberg San Francisco General Hospital in California, the United States, has eight positions when fully staffed – three trauma registrars, four performance improvement nurses, and one per diem nurse, whose tasks include performing chart review, data entry, coding, and abstraction from the electronic health record for the trauma registry, and tracking patient complications and issues. The goal is for concurrent data abstraction, entering the patient on the workday following patient hospital arrival and completing the expected registry entry within 60 days of patient discharge. Approximately 3700 patients are entered into the registry annually. The American College of Surgeons Committee on Trauma sets a standard of one full-time person for every 400-600 patient registry entries.^14^ It is unjust to expect frequently overworked, underpaid, and under supported clinical staff to take on this additional duty. Even if initially bolstered by external funds and teams, there must eventually be adequate support and funding for these efforts from within a country’s healthcare system, whether at the hospital, regional, or national level. As has been exhibited repeatedly, countries cannot afford to ignore preventable pathologies significantly impacting their citizens, such as with the ongoing, concomitant opioid crisis. Injuries are no different.

 As technology in the 21st century progresses, we must find ways to leverage it for equitable disease surveillance and intervention impact measurement, while minimizing added human effort and cost. For registries this could be done via free or low-cost electronic medical record platforms, like Open Medical Record System,^15^ affordable tablets and computers, and subsidized internet connectivity, the possibility for which is evidenced by its ubiquity with 5 billion internet users worldwide and a global penetration rate of 63%.^16^ We must move towards automating surveillance data collection across pathologies as is feasible over creating limitless separate registries that rely on data entry and multiple staff salaries to perform. We need to set pragmatic, context-relevant goals that prioritize progress over perfection. While the gold standard can remain striving for granular data that represent the population of interest, much can be done with select data from one or a few sites. Although comparisons between sites and countries for benchmarking can be helpful, the priority should first be to focus locally, building the foundation for hospital performance and quality improvement programs. Barthélemy et al must be commended on bringing the critical issue of neurotrauma and its accurate measurement to the forefront; however, excluding single hospital and regional registries reinforces an obsession with an ideal that prevents instead of augments progress. We must lead with inclusivity, as well as adaptability, taking what works in other locations and changing it for the local context, to avoid getting stuck at the struggle to perfectly measure the magnitude of the problem. These are important questions to consider that will require multidisciplinary, creative interventions likely occurring in parallel at all phases of the public health approach as we aim to impact the inordinate burden of neurotrauma and injuries globally. We must continue to work towards zero preventable deaths so everyone can live their lives to the fullest irrespective of where they live. Injuries are not inevitable. They have been the cause of a global pandemic for decades; one we cannot afford to ignore any longer.

## Ethical issues

 Not applicable.

## Competing interests

 Authors declare that they have no competing interests.

## Authors’ contributions

 Conception and design: MAB; drafting of the manuscript: MAB; critical revision of the manuscript for important intellectual content: MAB, OCK, and HS.
